# Rare PMP22 variants in mild to severe neuropathy uncorrelated to plasma GDF15 or neurofilament light

**DOI:** 10.1007/s10048-023-00729-5

**Published:** 2023-08-22

**Authors:** Edouard Palu, Julius Järvilehto, Jana Pennonen, Nadine Huber, Sanna-Kaisa Herukka, Annakaisa Haapasalo, Pirjo Isohanni, Henna Tyynismaa, Mari Auranen, Emil Ylikallio

**Affiliations:** 1 Department of Clinical Neurophysiology, Medical Imaging Center, Helsinki University Hospital, Helsinki, Finland; 2 Stem Cells and Metabolism Research Program, Faculty of Medicine, University of Helsinki, Helsinki, Finland; 3 A.I. Virtanen Institute for Molecular Sciences, University of Eastern Finland, Kuopio, Finland; 4 Department of Neurology, Kuopio University Hospital, Kuopio, Finland; 5 Neurology, Institute of Clinical Medicine, University of Eastern Finland, Kuopio, Finland; 6 Child Neurology, New Children’s Hospital, Pediatric Research Center, University of Helsinki and Helsinki University Hospital, Helsinki, Finland; 7 Clinical Neurosciences, Neurology, Helsinki University Hospital, Biomedicum Room 525B, Haartmaninkatu 8, 00290 Helsinki, Finland

**Keywords:** Charcot-Marie-Tooth disease, Biomarker, CMT1E, PMP22, Genetic neuropathy

## Abstract

Charcot-Marie-Tooth disease (CMT) is a heterogeneous set of hereditary neuropathies whose genetic causes are not fully understood. Here, we characterize three previously unknown variants in *PMP22* and assess their effect on the recently described potential CMT biomarkers’ growth differentiation factor 15 (GDF15) and neurofilament light (NFL): first, a heterozygous *PMP22* c.178G > A (p.Glu60Lys) in one mother-son pair with adult-onset mild axonal neuropathy. The variant led to abnormal splicing, confirmed in fibroblasts by reverse transcription PCR. Second, a de novo *PMP22* c.35A > C (p.His12Pro), and third, a heterozygous 3.2 kb deletion predicting loss of exon 4. The latter two had severe CMT and ultrasonography showing strong nerve enlargement similar to a previous case of exon 4 loss due to a larger deletion. We further studied patients with *PMP22* duplication (CMT1A) finding slightly elevated plasma NFL, as measured by the single molecule array immunoassay (SIMOA). In addition, plasma GDF15, as measured by ELISA, correlated with symptom severity for CMT1A. However, in the severely affected individuals with *PMP22* exon 4 deletion or p.His12Pro, these biomarkers were within the range of variability of CMT1A and controls, although they had more pronounced nerve hypertrophy. This study adds p.His12Pro and confirms *PMP22* exon 4 deletion as causes of severe CMT, whereas the previously unknown splice variant p.Glu60Lys leads to mild axonal neuropathy. Our results suggest that GDF15 and NFL do not distinguish CMT1A from advanced hypertrophic neuropathy caused by rare *PMP22* variants.

**Supplementary Information:**

The online version contains supplementary material available at 10.1007/s10048-023-00729-5.

## Introduction

Hereditary sensorimotor neuropathy or Charcot-Marie-Tooth disease (CMT) affects approximately 1:2500 persons and causes debilitating progressive distal sensory disturbance and muscle weakness [1]. Demyelinating CMT (defined as median or ulnar motor conduction velocity (MCV) in the forearm decreased below 38 m/s) is called CMT1, and axonal CMT, where median or ulnar MCV is > 38 m/s, is known as CMT2. The disease is so far incurable but advances in the understanding of genetic causes and disease mechanisms give promise for the development of pharmacologic treatments [2].

The genetics of CMT is still not fully known despite major advances in recent years [1, 3]. About 70% of CMT1 is caused by the heterozygous duplication of peripheral myelin 22 gene *PMP22*, denoted as CMT1A [4]. The reciprocal deletion of the same locus causes hereditary neuropathy with liability to pressure palsies (HNPP) [5]. Individuals with HNPP have heightened sensitivity to pressure-induced mononeuropathies particularly at vulnerable sites. In addition, there are missense variants, splice variants, small insertions, and deletions in *PMP22*. These variants are much rarer than *PMP22* duplication or deletion, accounting for 1–6% of all CMT1 [6, 7]. Such rare *PMP22* variants have been linked to CMT1 (denoted CMT1E when caused by *PMP22* variants other than duplication), HNPP, or Dejerine-Sottas syndrome (DSS). The clinical severity of rare *PMP22* variants displays large variability [6].

PMP22 constitutes 2–5% of peripheral myelin protein, and its mutant forms or excessive amounts of it induce misfolding, leading ER folding mechanisms or proteasomes to be overwhelmed, which activates unfolded protein responses (UPR) [8–11]. Conversely, a decreased amount of PMP22 protein in HNPP, whether caused by gene deletion or loss-of-function variants, leads to myelin dysfunction, predisposing to current leakage from axons and vulnerability to mechanical challenges [6]. In accordance with the protein’s high expression in Schwann cells, most *PMP22* variants are known to cause demyelination, but at least one case of axonal neuropathy caused by a single nucleotide variant in *PMP22* has been reported [12]. More information is needed on the spectrum of variants and genotype–phenotype correlations for *PMP22*.

Sensitive biomarkers for CMT are in demand in particular due to its slow progression rate [13]. Ultrasonographic nerve cross-sectional area (CSA) increases because of nerve hypertrophy in CMT1A [14]. Nerve CSA may reflect clinical severity of CMT1A [15], but its usefulness as a longitudinal biomarker has been questioned [16]. Of blood biomarkers, growth differentiation factor 15 (GDF15) was recently found elevated in serum of individuals with CMT1A and other subtypes of CMT [17, 18]. Other promising serum or plasma biomarkers include transmembrane protease serine 5 [19], certain microRNAs [20], neural adhesion molecule 1 [17], and neurofilament light chain (NFL) [21], although without confirmation in longitudinal studies [22]. Expression of GDF15 is responsive to integrated stress response [18], while NFL is a structural component of neurons that is released upon axon degeneration [23]. Rare variants in *PMP22* that cause severe CMT1E or DSS, such as exon 4 deletion [24] and point mutations [25, 26], induce protein misfolding and ostensibly a strong stress response with commensurate nerve enlargement [24]. However, whether the severe forms of disease correlate with a further elevation of these blood biomarkers is not known.

The objective of the current study is to characterize three rare *PMP22* variants causing disease of highly variable severity, and to analyze the effect of the variants on plasma biomarkers together with detailed genotype–phenotype assessment. Our results expand the mutational spectrum of *PMP22* and give new knowledge of the behavior of plasma biomarkers in rare severe CMT1E.

## Methods

### Standard protocol approvals, registrations, and patient consents

This study was approved by the institutional ethics review board of Helsinki University Hospital (decision number Asianro HUS/3280/2018). All participants gave written informed consent to the study.

### Patient recruitment and biomarker analyses

We recruited four individuals with previously unknown *PMP22* variants (individuals A-1, A-2, B, and C). For blood biomarker analyses, we recruited nine individuals with demyelinating CMT1A due to *PMP22* duplication, and two individuals with the axonal CMT2K who were verified carriers of *GDAP1* p.His123Arg. For nerve ultrasound, a further five individuals with CMT2K and five with CMT1A were recruited. Individuals with axonal CMT2 were selected as controls as marked nerve enlargement in axonal polyneuropathy is not expected [27, 28], while in CMT1A, nerves are markedly and diffusely enlarged [29].

Participants were investigated at the Helsinki University Hospital. Study size was determined by the prevalence of rare previously unreported PMP22 variants in the study center.

Disease severity was rated using the CMT neuropathy score (CMTNS) [30], or CMT examination score (CMTES), which excludes the last two items that are based on nerve conduction measurements [31]. Nerve conduction studies (NCS) and nerve ultrasound studies were all performed by the same clinical neurophysiologist with experience in neuromuscular ultrasound (E.P). NSC were done with Dantec Keypoint Focus, Natus Medical Incorporated. Nerve ultrasound was done with Samsung Medison RS 80A (18 MHz, linear array). Each nerve was identified with ultrasound, the angle of probe was adjusted so that the smallest cross-section was obtained, and the cross-sectional area was measured using the trace function of the ultrasound device to mark the inside of the hyperechoic border of each nerve. During measurement, compression on the nerve was avoided. The measurements were performed avoiding typical impingement sites at sites described by Grimm et al., which also provided reference values [32]. The CSA was measured in the ulnar nerve in the mid upper arm and mid forearm, in the median nerve in mid upper arm, the elbow and mid forearm, in the superficial radial nerve and in the posterior interosseus nerve at the arcade of Frohse, and in the tibial nerve and peroneal nerve at the popliteal fossa, the tibial nerve behind the medial malleolus, the superficial peroneal nerve above the lateral malleolus, and the sural nerve behind the lateral malleolus. The vagus nerve was measured at the carotid bulb. All measurements were performed on the right side.

Plasma GDF15 was measured with ELISA as described previously [33]. Plasma NFL and glial fibrillary acidic protein (GFAP) levels were quantified with the Quanterix single molecule array (Simoa, Billerica, MA, USA) HD-X analyzer using the Neuro 2-Plex B kit (ref# 103,520) according to the manufacturer’s instructions.

Control plasma was provided by Helsinki Biobank from anonymous individuals with criteria of similar age and sex distribution as our patients and exclusion of neurological disease diagnosis (ICD-10 code beginning with G). Recruitment time for this study was 2019–2022. Statistical analyses were performed with GraphPad Prism 9 using unpaired *t* test with Welch’s correction and simple linear regression.

### DNA sequencing

For individuals A-1 and A-2, Sanger sequencing of *PMP22* was performed at the hospital diagnostic service. The DNA of individual B was studied first by whole exome sequencing (WES) as described previously [34] and later by gene panel sequencing using the neuropathy comprehensive panel NGS-086.02 (Medizinish Genetisches Zentrum, Munich, Germany). We determined the exact location of deletion encompassing *PMP22* exon 4 by Sanger sequencing using the primers TTGAGGAAGGAAGCTAAAGTCTT and TTCTTAGCACATCAGGGCCA. The DNA of individual C was analyzed using Charcot-Marie-Tooth Neuropathy Panel Plus (Blueprint Genetics, Helsinki, Finland).

### Patient fibroblasts and reverse transcription PCR

Fibroblast cultures were established from skin biopsies. To inhibit nonsense-mediated decay, we treated the cells with 100 ng/µl cycloheximide for 24 h or left untreated. After this, we extracted RNA, synthesized cDNA, and performed PCR to identify the inclusion or exclusion of *PMP22* exon 3 using the primers TGCTGCTGTTCGTCTCCA and CAGCACTCATCACGCACAG.

## Results

### Novel PMP22 point mutation affecting splicing in individuals A-1 and A-2

Individual A-1 is a male who had normal early development. Around age 19, he noticed persistent numbness in his hands and pain in his neck and shoulders after carrying heavy burdens. Later, the numbness spread also to his chest. At first clinical assessment, he had sensory impairment in the anterior aspects of the upper limbs, which improved over several months. His clinical examination was otherwise unremarkable. Over the years, his symptoms progressed. At age 31, he had mild numbness around his right ankle and in his fingers but no other symptoms. NCS at age 31 showed normal motor and sensory NCVs in upper and lower extremities, whereas sensory amplitudes were decreased and tibial H-reflex was prolonged. Needle EMG showed mild chronic neurogenic changes in lower limbs. These changes were interpreted as mild sensorimotor axonal neuropathy. Nerve ultrasound showed no changes in nerve diameters. His CMT neuropathy score was 3.

The mother of A1, individual A-2, first came for a neurologic examination at age 46. She had had intermittent sensory loss and numbness in her fingers and toes for two years, usually lasting about two days at a time, and persistent intense pain and numbness in her feet after a strenuous walking trip. At her exam at age 57, her muscle strengths were excellent; she reported sensory loss in toes and had decreased vibration sense at ankles. NCS at age 57 showed decreased sensory amplitudes in the lower extremities and mildly prolonged H-reflex latencies. Nerve conduction velocities were normal except for mild focal slowing of the right ulnar nerve in the elbow. Needle EMG showed mild increase of motor unit size in the right tibial muscle. These findings were interpreted as mild sensorimotor distal axonal neuropathy. Peripheral nerve diameters were normal. Her CMT neuropathy score was 3.

**Fig. 1:**
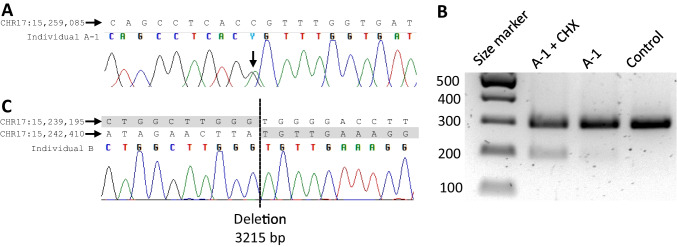
Sequencing reveals rare PMP22 variants. Sanger sequencing of individual A-1 (**A**) confirmed the variant c.178G > A (p.Glu60Lys, arrow). We then isolated RNA from skin fibroblasts and performed reverse transcription PCR with primers that produce a 291 bp product from the *PMP22* cDNA if exon 3 is included and a 191 bp product if exon 3 is excluded from the transcript. The 191 bp transcript was clearly visible when patient A-1’s fibroblasts were treated with nonsense-mediated decay inhibitor cycloheximide (CHX), and faintly visible in the untreated patient cells, but absent from the control cells (**B**). In individual B, we used Sanger sequencing to confirm the presence of a 3215 bp deletion that removes exon 4 (**C**)

After the confirmation of normal *PMP22* gene copy number in individual A-1, diagnostic Sanger sequencing revealed a previously unknown heterozygous variant c.178G > A predicting the p.Glu60Lys amino acid change (Fig. 1A). The same variant was present in his mother. This variant is absent from the Genome Aggregation Database (gnomAD v2.1.1). The variant changes a conserved amino acid residue and receives a CADD score of 32. In addition, as it is located in the last nucleotide of *PMP22* exon 3, we hypothesized that it affects the splicing of the gene. In silico prediction tool SpliceAI [35] gave a splice donor loss probability of 0.75. To test this, we obtained skin fibroblasts from individual A-1 and performed reverse transcription PCR with or without prior treatment of the fibroblasts with nonsense-mediated decay inhibitor cycloheximide. The control cells yielded the expected 291 bp PCR product corresponding to the wild-type allele. In A-1 fibroblasts treated with cycloheximide, we additionally observed a faint band of molecular size 191 bp, which corresponded to the predicted splice variant lacking exon 3 (length 100 bp) (Fig. 1B). The same 191 bp band was also very faintly visible in the untreated cells of individual A-1 (Fig. 1B). These results show that the variant alters splicing, leading to nonsense-mediated mRNA decay.

### Exon 4 deletion in individual B

Individual B came for evaluation due to clumsiness and delayed early motor development as a boy. He learned to walk at the age of 16 months. When examined at the age of 3 years and 10 months, he had hyperextending knees and slightly elevated foot arches, for which supporting insoles were prescribed. His condition was progressive, with increasing limb weakness and joint deformities. He required surgeries for foot deformities and for neurogenic scoliosis at age 14. Since his early teenage years, he has needed help in activities of daily living. His cognitive ability is normal. His latest clinical evaluation was done at age 24. His cranial nerve examination was normal. He was able to support his head independently and produced some movement to his shoulders. There was no movement in distal parts of the upper extremities; elbow flexion strengths were 1/5 and extension strengths 2/5 on both sides. Lower limbs were completely without movement. His CMT neuropathy score was 36.

**Fig. 2:**
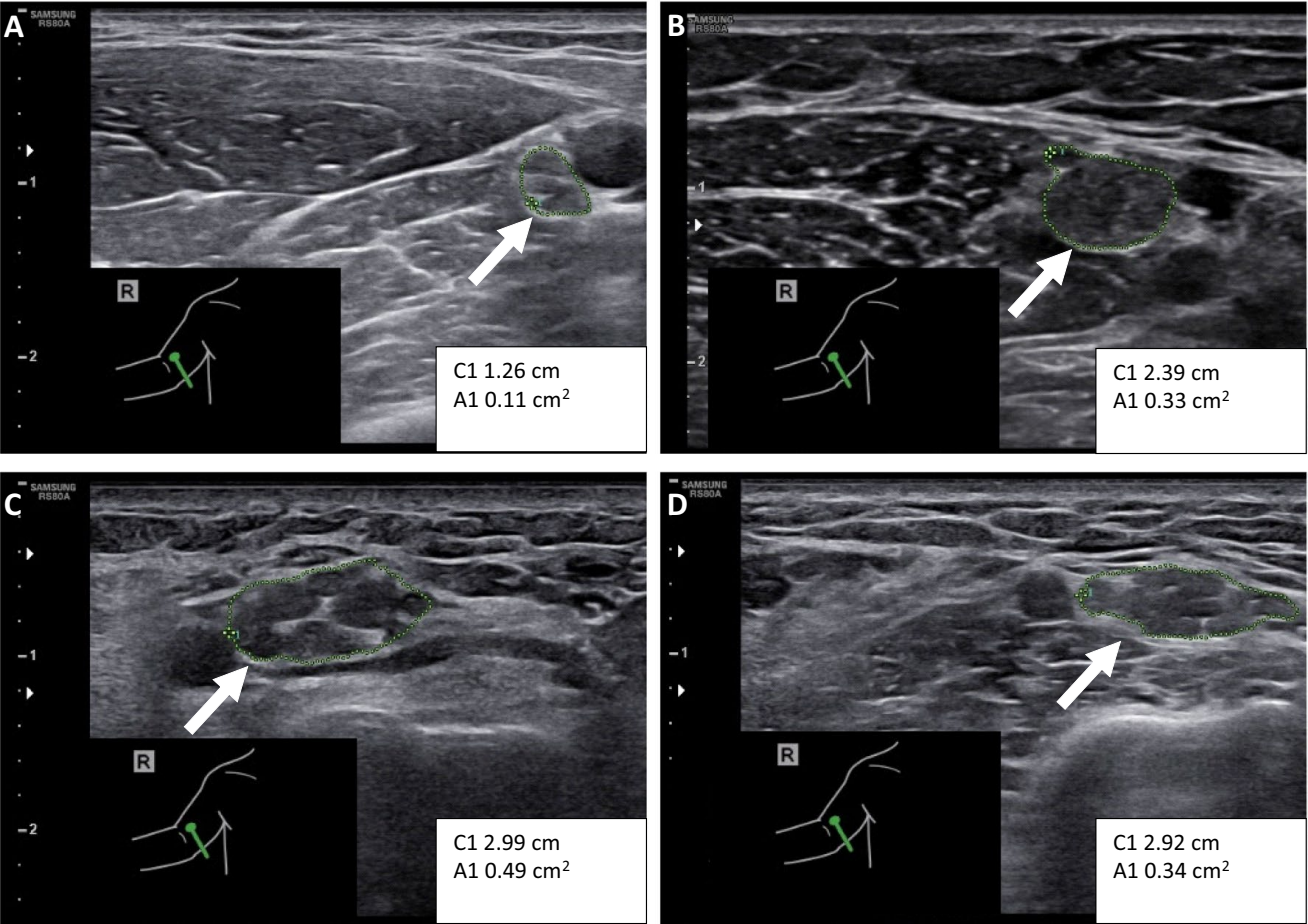
Extreme nerve enlargement in CMT1E. We investigated the grade of nerve enlargement in different subtypes of CMT. Shown are representative images of the median nerve at the upper arm (arrows and dotted lines). Nerve CSA was 11 mm^2^ in and individual with CMT2K (**A**) and 33 mm^2^ in an individual with CMT1A (**B**). For individual B with *PMP22* exon 4 deletion, the CSA was 49 mm^2^ (**C**) and for individual C with p.His12Pro variant, the CSA was 34 mm^2^ (**D**) in this location. *C1* circumference, *A1* area

His first NCS at age 3 was consistent with demyelinating polyneuropathy. Motor responses from the median and peroneal nerves were low in amplitude, conduction velocities very slow, and distal latencies severely prolonged. Unusually high stimulation intensities were necessary to elicit responses, and the study was interrupted without attempting to record sensory responses because of patient discomfort. At age 11, motor and sensory responses in upper and lower extremities were unobtainable except for a very low-amplitude motor response from the right median nerve with severely prolonged distal latency. At age 23, all motor and sensory responses were unobtainable; nerve ultrasound showed extreme thickening of the nerves, most prominently in the upper extremity (Fig. 2C) and the vagus nerve. Needle EMG suggested both chronic and acute axonal damage.

Individual B was the only affected person in his family. Genetic studies first excluded *PMP22* duplication and point mutations, and point mutations in *MPZ* and *GJB1*. WES revealed no suspected pathogenic changes. Finally, gene dosage analysis of a commercial gene panel suggested a heterozygous deletion involving *PMP22* exon 4. We were able to estimate the exact position of the deletion by identifying a split read in the implicated area from the previously generated WES data. We then performed PCR and Sanger sequencing of this region and found a deletion of 3215 bases (del_chr17:15,239,205–15,242,419; GRCh38/hg38) that includes the entire exon 4 (Fig. 1C) but is significantly smaller than the previously described disease-associated 17 kb deletion that includes exon 4 [36].

### PMP22 point mutation in individual C

Individual C is a female who had delayed motor development since infancy. Brain MRI and EEG at age 2 were normal. She was re-examined at age 7 because of clumsiness, falls, and reduced fine motor skills. At this time, NCS showed neuropathy with sensory and motor responses unobtainable at standard recording sites, and sural nerve biopsy showed demyelinating neuropathy with onion-bulb formation. Her condition was progressive. When last examined at age 25, she walked independently with ankle supports. She had atrophy of intrinsic hand muscles and considerable difficulty handling small objects. Distal muscle weakness was present in all limbs. Pinprick sensation was decreased from the knees onwards while sensation to vibration was absent in the lower limbs and reduced distal to the elbows. Her CMT neuropathy score was 24.

In NCS at age 24, we obtained similar results in that all responses were unobtainable at standard sites; however, using high stimulation intensities, a low-amplitude radial motor response was obtained from the extensor indicis muscle with extremely slow conduction velocity (2.8 m/s (sic); normal > 49 m/s). These findings suggest a severe demyelinating process. Nerve ultrasound showed extreme thickening of the nerves, most prominently in the upper extremity (Fig. 2D) and the vagus nerve.

Her family history was negative for neuropathy. The *PMP22* duplication was excluded. Gene panel revealed heterozygous *PMP22* c.35A > C, p.His12Pro variant, which was absent in both parents and gnomAD v2.1.1 and received a CADD score of 29.9.

The clinical features of all individuals are summarized in Table 1.

**Table 1:** Clinical features of individuals with previously unknown *PMP22* variants

Individual	Sex	PMP22 variant (all heterozygous)	AAO	AAE	CMTNS	Plasma GDF15 (pg/ml)	Plasma NFL (pg/ml)	Plasma GFAP (pg/ml)	Median nerve cross-section (upper arm, mm^2^) (mm^2^)	Motor NCS				Sensory NCS (antidromic)			
										Median		Radial		Ulnar		Sural	
										CMAP (mV) (LLN 4.0)	MCV (m/s) (LLN 49)	CMAP (mV) (LLN 2.0)	MCV (m/s) (LLN 49)	SNAP (µV) (LLN 17)	SCV (m/s) (LLN 50)	SNAP (µV) (LLN 6)	SCV (m/s) (LLN 40)
A-1	M	p.Glu60Lys	19	31	3	241	6.7	27	10	9.1	56	–	–	**5.0**	53	**1.3**	42
A-2	F	p.Glu60Lys	44	57	3	484	14	110	10	6.7	56	–	–	**13**	51	**abs**	**abs**
B	M	Deletion exon 4	3	24	36	447	11	132	49	**abs**	**abs**	**abs**	**abs**	**abs**	**abs**	**abs**	**abs**
C	F	p.His12Pro	0	25	24	416	15	63	34	**abs**	**abs**	**0.28**	**2.8**	**abs**	**abs**	**abs**	**abs**

*AAO* age at onset (refers to earliest record of symptoms documented by healthcare professional), *AAE* age at examination, *abs*. absent action potential, *CMTNS* Charcot-Marie-Tooth neuropathy score (scale 0–36), *CMAP* compound motor action potential, *GDF15* growth differentiation factor 15, *GFAP* glial fibrillary acidic protein, *MCV* motor conduction velocity, *NFL* neurofilament light, *SCV* sensory conduction velocity, *SNAP* sensory nerve action potential, *LLN* lower limit normal. Abnormal NCS are shown in bold

### Severe CMT1E is distinguished from other CMT by nerve enlargement but not GDF15 or NFL

**Fig. 3:**
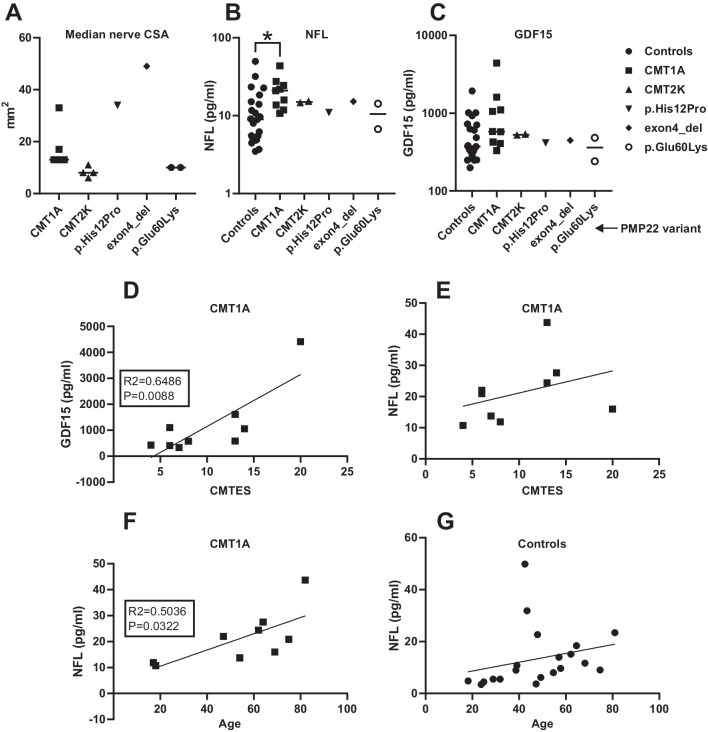
Severe CMT1E cases have similar blood biomarker levels as other CMT. Median nerve cross-section area (CSA) was higher in individuals with PMP22 p.His12Pro or exon 4 deletion (exon4_del) than in individuals with CMT1A or CMT2K (**A**). Of the plasma biomarkers, individuals with CMT1A had higher mean NFL (unpaired *t* test with Welch’s correction *P* = 0.0456) (**B**) and no significant change in mean GDF15 (**C**) when compared to controls without neurological disease. Individuals with CMT2K or the rare *PMP22* variants p.His12Pro, exon 4 deletion or p.Glu60Lys had NFL (**B**) and GDF15 (**C**) levels that were in the range of variation for individuals with CMT1A or controls. In individuals with CMT1A, the Charcot-Marie-Tooth examination score (CMTES) correlated significantly with plasma GDF15 (*R*2 = 0.6486, *P* = 0.0088, simple linear regression) (**D**), but showed no significant correlation to plasma NFL concentration (**E**). Furthermore, when compared to age, we found a significant correlation (*R*2 = 0.5036, *P* = 0.0322, simple linear regression) for the individuals with CMT1A (**F**) but no significant correlation for controls (**G**)

We measured the nerve CSA from the median nerve at the upper arm from the individuals with rare *PMP22* variants described in this study, and individuals with CMT1A or CMT2K. The severely affected individuals B (49 mm^2^) and C (34 mm^2^) had CSA above the range of variability for individuals with CMT1A (13–33 mm^2^), which in turn was larger than the range for individuals with CMT2K (6–11 mm^2^) (Fig. 3A; Table 1; Supplementary Table 1). All individuals also underwent NCS, where findings were consistent with axonal neuropathy for CMT2K and demyelinating neuropathy for CMT1A patients (Supplementary Table 2).

Next, we measured plasma GDF15, NFL, and GFAP levels in all groups of affected individuals and anonymous controls (Table 1; Supplementary Table 3). Individuals with CMT1A had higher mean plasma NFL than controls (*P* = 0.046, Fig. 3B), but for GDF15, we did not find a statistically significant change (Fig. 3C). However, when taking into account the CMT1A disease severity based on CMTES, we found that plasma GDF15 correlated with disease severity (simple linear regression, *R*2 = 0.4727, *P* = 0.0280, Fig. 3D) whereas we could not demonstrate the same for NFL (Fig. 3E). It must be noted that the median age of CMT1A patients (59 years ± 20 years S.D.) was higher than that of controls (48 years ± 17 years), and the levels of NFL correlated significantly with the age in individuals with CMT1A (Fig. 3F) but not in controls (Fig. 3G).

Individuals A-1, A-2, B, and C with previously unknown *PMP22* variants had comparable plasma GDF15 and NFL levels to individuals with CMT1A (Fig. 2A). GFAP was not significantly changed in affected individuals with CMT1A (168 pg/ml ± 87 pg/ml, mean ± S.D.) compared to controls (111 pg/ml ± 186 pg/ml), and the individuals with rare PMP22 variants were within the range of variability of individuals with CMT1A and controls (Table 1; Supplementary Table 3).

These findings are consistent with the increased plasma NFL in CMT1A, and plasma GDF15 positively correlated with the disease severity in CMT1A. However, for the rare *PMP22* variants, NFL or GDF15 was not elevated further despite severe disease and nerve enlargement in individuals B and C.

## Discussion

Here we have discovered three *PMP22* variants: p.Glu60Lys, p.His12Pro, and a 3215 bp deletion (del_chr17:15,239,205–15,242,419) that includes exon 4. Rare *PMP22* variants have been previously associated with a range of phenotypes, including CMT-type neuropathy denoted as CMT1E, HNPP, and severe congenital neuropathy known as DSS [37]. Exon 4 deletion has been reported previously in one sibling pair, then as part of a larger 17 kb deletion [24, 36]. Our results expand the spectrum of rare *PMP22* mutations and exemplify their genotype–phenotype correlations and their effects on plasma biomarkers.

The c.178G > A (p.Glu60Lys) variant was associated with a very mild phenotype. Both the mother (A-2) and son (A-1) of this family reported transient symptoms of numbness, pains, and weakness in different parts of the body. Clinically, the phenotype could be described as a very mild pressure-sensitive neuropathy; however, there was no clear neurophysiologic evidence of multifocal slowing of NCV as would be expected in HNPP. Both individuals had normal NCV examinations while sensory amplitudes were decreased, which together with neurogenic EMG findings were interpreted as axonal neuropathy. In comparison, the *PMP22* mutation p.Thr118Met causes HNPP in the heterozygous state and severe axonal neuropathy in the homozygous state [38]. Furthermore, the variant p.Arg159Cys was associated with axonal neuropathy [12]. Therefore, our results further support the need to consider rare *PMP22* mutations also when NCS suggests an axonal neuropathy.

We confirmed that the c.178G > A (p.Glu60Lys) variant affects splicing, which leads to a frame-shift and loss of RNA through nonsense-mediated decay. Bellone et al. [39] reported a heterozygous G > A transversion at nucleotide c.179 + 1 located at the 5’ donor splice site of intron 2. This variant led to a splicing defect, the production of an abnormal mRNA containing a fragment from intron 2 that predicted a premature stop and an HNPP phenotype [39]. Other intronic splice site mutations have been reported at c.78 + 1 [40], c.179-1G > C [41, 42], c.78 + 5G > A, c.320-1G > C [43], and c.319 + 1 [42]. These variants are likely to prevent the production of functional protein, but all have not been tested functionally. Therefore, the result of the c.178G > A variant is likely to be a decreased amount of PMP22 protein. The expected clinical consequence of PMP22 haploinsufficiency is HNPP. As our patient’s phenotype differs from typical HNPP, we cannot exclude the presence of modifying factors. For instance, a small amount of abnormally spliced transcript may be able to escape nonsense-mediated decay, or a small amount of mutant pre-mRNA may be able to splice normally, allowing the production of protein with the p.Glu60Lys change. Given its location in the extracellular loop of PMP22, this variant could affect the interaction with myelin protein zero (MPZ) [44], another gene linked to both axonal and demyelinating phenotypes. The disturbed interaction of PMP22 and MPZ could then give rise to additional detrimental effects from the protein produced by a small amount of correctly spliced mRNA.

The 3.2 kb deletion including *PMP22* exon 4 deletion caused a severe disease with a loss of ambulation in individual B. De novo p.His12Pro variant appeared somewhat less detrimental for individual C. Both individuals had severe nerve hypertrophy, which we documented on an ultrasonography, suggesting a strongly activated pathologic process. Likely causes of nerve CSA increase are collagen deposition and Schwann cell hyperplasia, which do not necessarily correlate with disease severity as they do not reflect the degree of axonal loss. Nerve CSA was larger than in axonal CMT2K or even in CMT1A, where nerve CSA is known to be enlarged [14]. The previously reported pair of sisters with a 17 kb deletion of *PMP22*, which also led to the exclusion of exon 4, had similar severe early-onset disease, although the older sister appeared somewhat less severely affected than individual B as she was able to walk with orthoses at age 15 [24]. The *PMP22* exon 4 deletion differs from most other known indel or splice variants because it produces an in-frame change and therefore does not lead to mRNA instability. Interestingly, loss of exon 4 leads to an aberrant protein, which is trapped in the ER [24]. p.His12 in turn is positioned in the transmembrane domain 1 (TM1). Other variants in the same position, p.His12Gln [45] and p.His12Arg [42], are known to cause diseases. Several other disease-causing point mutations are known in TM1, and these variants are also likely to induce folding defects [46–51]. Their likely effects on protein folding combined with extreme clinical severity suggest that exon 4 deletion and p.His12Pro may cause a strong unfolded protein response, which hypothetically could cause even higher GDF15 elevation than what is typical in CMT1A.

GDF15 was recently shown to have strong potential as a biomarker for CMT [17], in addition to cardiometabolic diseases [52]. Plasma NFL in turn is a marker of axonal degeneration, which is elevated in several neurological diseases including CMT. In our individuals with CMT1A, we found slight elevation of plasma NFL, and GDF15 correlated with symptom severity. It must be noted that NFL elevation was only borderline significant, and there was variability in both GDF15 and NFL levels. For controls, we used biobanked samples excluding neurological disease, but we cannot exclude the presence of cardiovascular disease or undiagnosed neurological disease that could have produced elevated levels in some of the controls. Furthermore, plasma NFL increases with increasing age [53] and GDF15 is upregulated during aging [54]. A small difference in average age between the groups may therefore have affected the comparison of CMT1A to controls. Thus, no definitive conclusions regarding its usefulness as a biomarker for CMT1A can be made based on this relatively small sample set, but the sample is useful for the comparison of rare cases of advanced CMT1E to CMT1A.

The severe demyelinating phenotypes of individuals B and C were not reflected in GDF15 or NFL levels, which were comparable to those of other CMT patients. This could imply a ceiling effect for GDF15 and NFL such that further severity of a hypertrophic neuropathy does not cause additional elevation of the biomarker. On the other hand, we cannot exclude that GDF15 and NFL could be more significantly elevated early in the course of their disease, when disease progression tends to be faster and neurodegeneration may have been more active. GFAP in turn has been suggested as a marker for multiple sclerosis and traumatic brain injury [55] but based on our results does not respond to peripheral nervous system demyelination. The most important limitations of this study are a small sample size and lack of longitudinal biomarker measurements. Further studies are warranted on the behavior of plasma biomarkers in rare PMP22-related neuropathy. Ideally, biomarkers should be measured at the time of peak clinical progression change such as during puberty years.

In summary, this study presents the genetic and clinical characterization of three *PMP22* variants. The dominant p.Glu60Lys splice altering variant is important as it confirms the possibility of axonal findings in *PMP22*-related disease. The de novo exon 4 deletion and p.His12Pro further expand the genetic spectrum of severe demyelinating neuropathy, for which the degree of nerve hypertrophy can correlate with clinical symptoms.

### Supplementary Information

Below is the link to the electronic supplementary material.

Supplementary file1 (DOCX 31 KB)

## Data Availability

The datasets generated and/or analyzed during the current study are available from the corresponding author on reasonable request.
