# Supplementary material for: Rare PMP22 variants in mild to severe neuropathy uncorrelated to plasma GDF15 or neurofilament light
Source: Neurogenetics. 2023 Aug 22;24(4):291–301. doi: 10.1007/s10048-023-00729-5 (PMC10545620; doi:10.1007/s10048-023-00729-5)
Supplement: Supplementary file 1 — Supplementary file1 (DOCX 31 KB) [file 10048_2023_729_MOESM1_ESM.docx]

| Individual | Diagnosis/ variant | Age | Sex | Height (m) | CMTNS | Nerve cross sectional area (mm^2^) | | | | | | | | | | | | |
| --- | --- | --- | --- | --- | --- | --- | --- | --- | --- | --- | --- | --- | --- | --- | --- | --- | --- | --- |
|  |  |  |  |  |  | Median  upper arm | Median elbow | Median forearm | Ulnar upper arm | Ulnar forearm | Superficial radial | Radial PIN | Tibialis poplitea | Tibialis ankle | Deep peroneal poplitea | Superifical peroneal ankle | Vagus | Sural ankle |
|  |  |  |  |  |  |  |  |  |  |  |  |  |  |  |  |  |  |  |
| 1 | CMT2K | 38 | F | 1.55 | 0 | 6 | 5 | 5 | 4 | 5 | 2 | 1 | 26 | 12 | 6 | 2 | 2 | 4 |
| 2 | CMT2K | 43 | F | 1.62 | 2 | 8 | 8 | 5 | 5 | 5 | 1 | 2 | 19 | 9 | 6 | 1 | 2 | 2 |
| 3 | CMT2K | 27 | M | 1.72 | 8 | 11 | 8 | 8 | 7 | 6 | 1 | 1 | 31 | 10 | 7 | 1 | 1 | 2 |
| 4 | CMT2K | 52 | F | 1.58 | 2 | 8 | 6 | 6 | 5 | 6 | 2 | 2 | 15 | 6 | 9 | 2 |  | 3 |
| 5 | CMT2K | 38 | M | 1.78 | 1 | 8 | 5 | 6 | 5 | 6 | 1 | 2 | 18 | 7 | 6 | 3 |  | 2 |
| 6 | CMT1A | 56 | M | 1.87 |  | 13 | 17 | 7 | 9 | 7 | 2 | 2 | 15 | 10 | 16 | 3 | 3 | 4 |
| 7 | CMT1A | 46 | F | 1.77 | 11 | 13 | 17 | 9 | 17 | 8 | 2 | 3 | 38 | 8 | 11 | 3 | 3 | 4 |
| 8 | CMT1A | 41 | F | 1.67 | 17 | 17 | 16 | 10 | 10 | 8 | 2 | 2 | 35 | 21 | 11 | 4 | 4 | 3 |
| 9 | CMT1A | 51 | F | 1.75 | 12 | 13 | 9 | 13 | 19 | 12 | 2 | 2 | 49 | 22 | 15 | 3 | 2 | 5 |
| 10 | CMT1A | 19 | M | 1.72 | 11 | 33 | 20 | 29 | 18 | 16 | 5 | 4 | 62 | 30 | 21 | 4 | 4 |  |
| C | p.His12Pro | 24 | F | 1.60 | 24 | 34 | 20 | 15 | 30 | 12 | 2 | 2 | 36 | 6 | 27 | 2 | 4 | 3 |
| B | Deletion exon 4 | 23 | M | 1.70 | 36 | 49 | 43 | 33 | 40 | 19 | 6 |  | 67 | 18 | 17 | 7 | 9 | 5 |
| A-1 | p.Glu60Lys | 31 | M | 1.90 | 3 | 10 | 7 | 5 | 6 | 5 | 2 | 1 | 33 | 8 | 10 | 2 | 2 | 2 |
| A-2 | p.Glu60Lys | 57 | F | 1.63 | 3 | 10 | 8 | 9 | 8 | 6 | 2 | 2 | 30 | 10 | 11 | 2 | 2 |  |

**Supplementary table 1.** **Nerve cross-sectional area (CSA).** We measured nerve cross-section (CSA) with ultrasound are at multiple locations in individuals with CMT1A or CMT2K to compare with individuals A-1, A-2, B and C with rare PMP22 variants. Measurements were made from =the right side. In addition, here are shown Charcot-Marie-Tooth neuropathy scores (CMTNS) for the studied individuals. Empty=data not available.

**Supplementary table 2.** **Nerve conduction studies.** Shown are nerve conduction studies for individuals A-1, A-2, B and C with rare PMP22 variants as well as individuals with CMT1A or CMT2K to compare. Measurements were made from the right side. CMTNS = Charcot-Marie-Tooth neuropathy score (scale 0-36), CMAP = compound motor action potential, MCV = motor conduction velocity, SCV = sensory conduction velocity, SNAP = sensory nerve action potential, abs.=absent response. Empty=data not available.

| Individual | Diagnosis/ variant | Age | Sex | CMTNS | Motor NCS | | | | Sensory NCS | | | |
| --- | --- | --- | --- | --- | --- | --- | --- | --- | --- | --- | --- | --- |
|  |  |  |  |  | Ulnar (forearm) | | Median | | Ulnar antidromic | | Sural | |
|  |  |  |  |  | CMAP (mV) (LLN 6.0) | MCV (m/s) (LLN 49) | CMAP (mV)  (LLN 4.0) | MCV (m/s)  (LLN 49) | SNAP (µV)  (LLN 17) | SCV (m/s)  (LLN 50) | SNAP (µV)  (LLN 6) | SCV (m/s)  (LLN 40) |
| 1 | CMT2K | 38 | F | 0 | 9.0 | 61 | 4.5 | 61 | 18 | 61 | **5.2** | 54 |
| 2 | CMT2K | 43 | F | 2 | 6.3 | 61 | 5.6 | 61 | **5.5** | 54 | **2.9** | 43 |
| 3 | CMT2K | 27 | M | 8 | 7.7 | 55 | 4.3 | 51 | **abs.** |  | **1.6** | 41 |
| 4 | CMT2K | 52 | F | 2 | 6.3 | 55 | **3.2** | 55 | 17 | 69 | **4.9** | 40 |
| 5 | CMT2K | 38 | M | 1 | 6.4 | 60 | 6.2 | 56 | **6.8** | 57 | 7.4 | 58 |
| 6 | CMT1A | 56 | M |  | **2.7** | **30** | **0.31** | **26** | **abs.** |  | **abs.** |  |
| 7 | CMT1A | 46 | F | 11 | **3.6** | **33** | 4.1 | **28** | **1.1** | **27** | **abs.** |  |
| 8 | CMT1A | 41 | F | 17 | **3.4** | **23** | **2.8** | **27** | **4.0** | **25** | **abs.** |  |
| 9 | CMT1A | 51 | F | 12 | **2.3** | **30** | **2.6** | **31** | **2.9** | **20** | **abs.** |  |
| 10 | CMT1A | 19 | M | 11 | **2.1** | **14** | **2.0** | **16** | **5.9** | **19** | **abs.** |  |
| C | p.His12Pro | 24 | F | 24 | **abs.** |  | **abs.** |  | **abs.** |  | **abs.** |  |
| B | Deletion exon 4 | 23 | M | 36 | **abs.** |  | **abs.** |  | **abs.** |  | **abs.** |  |
| A-1 | p.Glu60Lys | 31 | M | 3 | 9.2 | 58 | 9.1 | 56 | **5.0** | 53 | **1.3** | 42 |
| A-2 | p.Glu60Lys | 57 | F | 3 | 7.8 | 58 | 6.7 | 56 | **13** | 51 | **abs.** |  |

**Supplementary table 3.** Blood biomarkers. We measured the levels of potential blood biomarkers of Charcot-Marie-Tooth disease (CMT), growth differentiation factor 15 (GDF15), neurofilament light (NFL) and glial fibrillary acidic protein (GFAP), from individuals with CMT1A or CMT2K in order to compare with the individuals A-1, A-2, B and C who had rare PMP22 variants. Shown are blood levels for each of the measured molecules together with the CMT examination score (CMTES).

| Individual | Diagnosis | Age | Sex | CMTES | GDF15 (pg/ml) | NFL (pg/ml) | GFAP (pg/ml) |
| --- | --- | --- | --- | --- | --- | --- | --- |
| 10 | CMT1A | 18 | M | 4 | 426 | 10.7 | 66.3 |
| 11 | CMT1A | 82 | M | 13 | 1608 | 43.7 | 323.9 |
| 12 | CMT1A | 64 | M | 14 | 1054 | 27.5 | 193.2 |
| 13 | CMT1A | 69 | F | 20 | 4412 | 16.0 | 110.3 |
| 14 | CMT1A | 47 | F | 6 | 407 | 22.0 | 61.9 |
| 15 | CMT1A | 62 | F | 13 | 580 | 24.4 | 203.9 |
| 16 | CMT1A | 75 | M | 6 | 1103 | 20.9 | 163.7 |
| 17 | CMT1A | 54 | F | 7 | 331 | 13.7 | 131.3 |
| 18 | CMT1A | 17 | F | 8 | 577 | 11.9 | 256.0 |
| 19 | CMT2K | 30 | F | 2 | 537 | 15.3 | 49.8 |
| 20 | CMT2K | 39 | M | 2 | 524 | 14.7 | 43.1 |
